# Rapid Differentiation of Unfrozen and Frozen-Thawed Tuna with Non-Destructive Methods and Classification Models: Bioelectrical Impedance Analysis (BIA), Near-Infrared Spectroscopy (NIR) and Time Domain Reflectometry (TDR)

**DOI:** 10.3390/foods11010055

**Published:** 2021-12-27

**Authors:** Sonia Nieto-Ortega, Ángela Melado-Herreros, Giuseppe Foti, Idoia Olabarrieta, Graciela Ramilo-Fernández, Carmen Gonzalez Sotelo, Bárbara Teixeira, Amaya Velasco, Rogério Mendes

**Affiliations:** 1 AZTI, Food Research, Basque Research and Technology Alliance (BRTA), Parque Tecnológico de Bizkaia, Astondo Bidea, Edificio 609, 48160 Derio, Spain; amelado@azti.es (Á.M.-H.); gfoti@azti.es (G.F.); iolabarrieta@azti.es (I.O.); 2 Instituto de Investigaciones Marinas, CSIC, Eduardo Cabello, 6, 36208 Vigo, Spain; graciela@iim.csic.es (G.R.-F.); carmen@iim.csic.es (C.G.S.); amayavelasco@iim.csic.es (A.V.); 3 Portuguese Institute for the Sea and Atmosphere, IPMA, R. Alfredo Magalhães Ramalho, 6, 1449-006 Lisbon, Portugal; barbara.p.b.teixeira@gmail.com (B.T.); rogerio@ipma.pt (R.M.); 4 Interdisciplinary Center of Marine and Environmental Research (CIIMAR), University of Porto, Rua das Bragas 289, 4050-123 Porto, Portugal

**Keywords:** chemometrics, water injection, fishery products, authenticity, sensors, defrosted, freezing, quality control, consumer trust, labelling

## Abstract

The performances of three non-destructive sensors, based on different principles, bioelectrical impedance analysis (BIA), near-infrared spectroscopy (NIR) and time domain reflectometry (TDR), were studied to discriminate between unfrozen and frozen-thawed fish. Bigeye tuna (*Thunnus obesus*) was selected as a model to evaluate these technologies. The addition of water and additives is usual in the fish industry, thus, in order to have a wide range of possible commercial conditions, some samples were injected with different water solutions (based on different concentrations of salt, polyphosphates and a protein hydrolysate solution). Three different models, based on partial least squares discriminant analysis (PLS-DA), were developed for each technology. This is a linear classification method that combines the properties of partial least squares (PLS) regression with the classification power of a discriminant technique. The results obtained in the evaluation of the test set were satisfactory for all the sensors, giving NIR the best performance (accuracy = 0.91, error rate = 0.10). Nevertheless, the classification accomplished with BIA and TDR data resulted also satisfactory and almost equally as good, with accuracies of 0.88 and 0.86 and error rates of 0.14 and 0.15, respectively. This work opens new possibilities to discriminate between unfrozen and frozen-thawed fish samples with different non-destructive alternatives, regardless of whether or not they have added water.

## 1. Introduction

Freezing and frozen storage has been widely used by the fish industry for extending the storage life of this perishable foodstuff. However, unfrozen seafood products are still highly demanded in most countries [1], having usually higher market prices than frozen ones [2]. Due to its high price and because in most cases consumers are unlikely to perceive visually the differences after thawing, unfrozen fish has been shown to be vulnerable to adulteration and fraudulent mislabeling involving replacement by frozen-thawed products [3,4].

The addition of water and additives in fish is also a common practice in the fish industry, which could be used together with the freezing and frozen storage to improve the quality of seafood products [5] and to avoid drip loss [6]. However, if the amount of added water is less than the 5% of the weight of the product, it is not necessary to be indicated on the label [7].

According to international regulations, frozen-thawed fish must be labelled as defrosted or previously frozen and must not be refrozen [7,8,9]. In addition, EU regulation No 1276/2011 [10] claims that food business operators placing on the market fishery products intended to be consumed raw must ensure that the product undergoes a freezing treatment, in order to kill parasites. For these reasons, there has been a need, within the food industry and/or official control authorities, to control whether the products for sale are really unfrozen or have been previously frozen and thawed [11]. However, accurate determinations are very difficult because their chemical and physical characteristics are very alike [12]. Official control reports of fraud are therefore scarce, and those available rely on qualitative subjective evaluations (e.g., muscle consistency, eye opacity, etc.) or need sophisticated laboratory equipment (e.g., for enzyme analysis, red blood cell analysis) [4].

Physical methods to discriminate between unfrozen and frozen-thawed fish are gaining wide attention due to their advantages (rapidness, on-site detection, portability and high accuracy) and have been the preferred method for the development of non-destructive analysis. The reported methods for this application were shown to be based mostly on magnetic resonance imaging [13], hyperspectral imaging [14], electric resistance of tissues [15,16] and spectroscopic techniques, such as electrical impedance [17], near- and mid-infrared [18,19], Raman [20] and fluorescence spectroscopy [21]. However, it is possible that some samples have added water and additives which are not claimed in the label. This could affect the discrimination between unfrozen and frozen-thawed samples by non-destructive methods if the technology selected for the detection is sensitive to changes in water content.

In this work, three technologies were studied to check their potentiality to differentiate between unfrozen and frozen-thawed fish. They are rapid, portable, non-destructive, and all of them have shown possibilities regarding the differentiation between unfrozen and frozen-thawed fish. Bioelectric Impedance Analysis (BIA) is based on the measurement of resistance (R), reactance (Xc) and phase angle (Pa) that are used to calculate the impedance of a tissue when a low intensity electrical current is passing through it. Some studies have determined that Xc and Pa exhibited differences between fresh and frozen fish, due to changes caused by the freezing process, such as cell membrane damage which, upon thawing, can cause intracellular fluid leakage, with consequent increased conductivity [22]. Near infrared spectroscopy (NIR) is a vibrational technique [23] which exploits the absorption of the light wavelengths in the electromagnetic spectrum between 780 nm and 2500 nm [24]. An incident radiation interacts with the tissues and returns physic-chemical information of the samples, in the form of a spectrum [25]. It has been used to differentiate between fresh and frozen-thawed samples in different seafood products such as tuna [26] or horse mackerel [27]. Finally, time domain reflectometry (TDR) is a methodology which probes the dielectric properties (DPs) of the material under test in a broad frequency range by measuring the voltage signal reflected by the sample as a function of time. Oscillating electric fields in the range of microwaves affect the vibrational dynamics of polar molecules such as water (the major constituent of food). Therefore, changes in the amount of water or aggregation state in the muscle are expected to be detected by this technology [28,29].

The objective of this work was to study the performance of these three non-destructive methods to differentiate between unfrozen and frozen-thawed tuna. To the authors’ knowledge, this is the first time that these sensors are studied together for this objective also considering the possibility that fish samples may have water and additives added, an important circumstance taking into account that BIA, NIR and TDR are technologies sensitive to water changes [29,30,31].

## 2. Materials and Methods

### 2.1. Sample Preparation

#### 2.1.1. Tuna Processing

Eleven dorsal and ventral bigeye tuna loins (*Thunnus obesus*) were used to carry out the experiment. Seven were purchased from a local supplier (Lisbon, Portugal) in July 2018 and four in July 2019, both coming from the FAO 34 fishing area (near Madeira Island, Portugal).

Tuna loins were cut in portions of about 500 g, obtaining 120 samples. Fifty tuna portions were randomly picked in 2018 and forty in 2019 and were manually injected with a 10% weight of five different water and additives solutions. The remaining 30 (10 from 2018 and 20 from 2019) were used as control. The water and additives solutions were as follows: (A) 3% salt; (B) 3% salt + 3% polyphosphates; (C) 3% salt + 5% polyphosphates; (D) 3% salt + 5% hydrolysate prepared at home from four-spot megrim *Lepidorhombus boscii*; and (E) Pescamine 150 (commercial polyphosphates blend). Those additives, which are typically used in fish industry practices, are commonly used to potentiate the water retention in the samples [32].

After the injections, tuna samples were covered with clinging film to avoid dehydration, and they remained 3 h at 3 ± 1 °C for stabilization. Before the freezing and thawing process, samples were vacuum packed.

#### 2.1.2. Freezing and Thawing Process

The freezing process was performed for 40 h at −20 °C, with an average freezing rate of 0.01 °C/min, so that the temperature in the center of the samples was −12 ± 1 °C. The objective was to reproduce the conditions of a possible imperfect freezing procedure, which is usually the case in seafood processors and restaurants. Afterwards, samples were thawed at 3 ± 1 °C.

#### 2.1.3. Additives and Reagents

The reagents used during the experiment, provided by Merck (Darmstadt, Germany), were of analytical grade. Regarding the additives, the salt (NaCl) used to prepare the solutions was of food grade. Pescamine 150 is a mixture of additives (E339, E450, E451, E452) composed of various types of phosphates and sodium phosphates and was prepared according to the manufacturer’s procedure (Vaessen-Schoemaker, Deventer, The Netherlands).

### 2.2. Destructive Analysis: Physicochemical Characterization

Physicochemical characterization consisted of the determination of moisture, protein and fat contents in thawed samples (control and injected samples). Moisture was analyzed also in unfrozen samples (control and injected). While protein and moisture were analyzed in all the samples in duplicate, the fat content was determined only in 8 control samples and in 4 injected samples per solution (20 samples in total), also in duplicate. Data were expressed in all the cases in percentages.

Crude protein content was analyzed in a LECO FP-528 protein/nitrogen determinator (LECO Corp, St. Joseph, MI, USA) by the Dumas combustion method [33], calibrating the equipment with ethylene diamine tetraacetic acid. Moisture content was determined according to the official gravimetric analysis [34]. Fat content was extracted with the Bligh and Dyer method [35] and determined by difference of weight. For the extraction, 2 g of minced tuna were homogenized in a Polytron homogenizer (10,000 rpm for 1 min) with methanol (4 mL) and dichloromethane (2 mL). Afterwards, another 2 mL of dichloromethane and 2 mL of water were added and homogenized again in the same conditions. Following that process, a centrifugation was performed (3000 rpm at 5 °C for 10 min). The organic phase was collected, and a second extraction was performed. The recovered organic phases were mixed and filtered with anhydrous sodium sulphate. The solvent was evaporated, leaving only the fat.

### 2.3. Data Acquisition

Data acquisition was adapted for each sensor, ensuring a homogeneous sampling with all of them. All the samples (unfrozen and thawed) were kept in the refrigerator before the analysis (3 ± 1 °C), so they were not influenced by temperature differences.

#### 2.3.1. Bioelectrical Impedance Analysis (BIA)

R, Xc and Pa measures were directly obtained with a bioimpedance analyzer BIA 101 Anniversary (BIA from now on) from Akern SRL (Pontassieve, Italy). The BIA device works by applying a high frequency (50 kHz) and very low amplitude (400 μA) electrical current through all the samples. A tetrapolar electrode was transformed into a dipolar electrode cable set by the manufacturer, which does not interfere with the results according to their experience. Later, the electrode pair was fixed to a PVC plate to keep a constant detector length distance (52 mm between the electrodes). Bioimpedance measurements were performed by inserting the electrode pair in the center of the external side of the loin, 1 cm deep into each tuna sample.

For the data acquisition with BIA, one measurement was performed per sample. All the unfrozen samples from both years (120) were measured before the injection with the different solutions. After the injection (50 samples in 2018 and 40 samples in 2019), measurements were performed again (N_unfrozen_ = 210). Afterwards, the freezing and thawing process in all the 120 samples was carried out. A total of 30 samples from 2019 (10 non-injected and 20 injected) were used for another experiment, and the remaining thawed samples were measured again (N_thawed_ = 90).

#### 2.3.2. Near-Infrared Spectroscopy (NIR)

A MicroNIR OnSite (Viavi, Italy) was used for NIR experiments. It is a portable and handheld device, working at a wavelength range from 900 to 1650 nm with a resolution of 6 nm. It has two integrated vacuum tungsten lamps and a 128-pixel InGaAs photodiode array (spectral resolution of <1.225%). The sensor was configured so that each spectrum was the average of 100 scans, using an integration time of 8.2 ms.

To determine NIR data, 8 scans at different points were acquired per sample, each scan being used as an independent measurement in the classification model. Again, all the unfrozen samples were measured before and after the injection (N_unfrozen_ = 1680 scans). After the freezing and thawing process, the samples were scanned again (N_thawed_ = 720 scans).

#### 2.3.3. Time Domain Reflectometry (TDR)

For TDR measurements, a patented smart system based on the principle of dielectric spectroscopy, namely, the RFQ-Scan^®^ (Radio Frequency Quality Scan) developed by Sequid GmbH (Bremen, Germany), was used. The device generates a step-like voltage signal of 2.56 ns (total duration) with a rise time of approximately 100 ps. The signal propagates through a coaxial cable till the open-end termination of the sensor where it interacts with the sample. The reflected signal in time domain is finally visualized and recorded. In the frequency domain, the signal is characterized by a broad bandwidth of approximately 5 GHz [36].

The acquisition was made as the average of 8 scans from different points per each sample. A total of 120 unfrozen tuna samples were scanned (30 non-injected and 90 injected) (N_unfrozen_ = 120) and 20 non-injected and 70 injected samples (N_thawed_ = 90) in thawed tuna.

A summary of the scans performed with each sensor is shown in Table 1.

### 2.4. Data Analysis

#### 2.4.1. Data Cleaning, Data Preprocessing and Principal Component Analysis (PCA)

The first step was to clean the data, that is, to eliminate samples with errors in the data acquisition. Then, a PCA model was developed (Matlab 2013a and PLS_toolbox version 8.2.1, The Matworks, Natick, MA, USA) in order to explore the data from the 3 sensors. Data were mean centered before the analysis.

After that, data from NIR and TDR were preprocessed with the objective of removing the physical artifacts and to improve the classification models [37]. Several pre-processing techniques were tested on data from both sensors: standard normal variate (SNV) with and without detrend, first and second derivatives of Saviztky–Golay (using different windows and polynomial orders) and combinations of all these techniques. After that, data were either mean centered or autoscaled (both techniques were tried). On the contrary, in BIA data, only mean center and autoscaling were tested before the creation of the models.

#### 2.4.2. Comparison between Unfrozen and Frozen-Thawed Samples

With the aim of observing the water loss in the samples during the frozen-thawed process, and to assure that samples still had added water once they were thawed, an ANOVA analysis was performed between the moistures of unfrozen and thawed samples. Afterwards, the moisture/protein ratio was calculated in control (non-injected) and injected thawed samples, performing another ANOVA with the objective of studying these differences. Both analyses were carried out using the software Statgraphics centurion XVI (Statgraphics Technologies, Inc., The Plains, VA, USA).

#### 2.4.3. Creation and Descriptive Statistics of Calibration and Validation Datasets

The Duplex Algorithm was used to create two datasets: one for calibration, with 80% of the data, and another for validation, with the remaining 20%. For that purpose, data from each sensor were standardized and orthonormalized, and the Euclidean distance between all pairs of points were calculated. The two points which had the biggest distance between them were assigned to the calibration set. In the remaining list, the next two points with the biggest distance were assigned to the validation set. It continues until every point was assigned in one set of samples [38].

The objective was to obtain two sets of balanced data for each technology (same proportion of unfrozen and frozen thawed and non-injected and injected samples in each). Furthermore, the aim of mixing samples from both years was to include information concerning spectral variance related to fishing years in both sets of samples in order to increase the robustness of the model. For that purpose, the algorithm was applied to the data obtained with each sensor, obtaining a different calibration and validation dataset for each technology (since the data acquisition was different in each case).

To ensure that calibration and validation datasets were well-balanced, a descriptive statistics study of the physicochemical data from both sets of samples was performed. The mean and typical deviation values were calculated, and after that, an ANOVA analysis was carried out to evaluate the differences between both sets. The analyses were carried out using the Statgraphics centurion XVI software.

#### 2.4.4. Classification Model Building

Classification models were developed using Matlab 2020b (The Matworks, Natick, MA, USA) coupled with the Classification toolbox (version 5.4) developed by Milano Chemometrics and QSAR Research group [39]. In this study, a model based on Partial Least Squares Discriminant Analysis (PLS-DA) was developed for each sensor. This is a linear classification method based on the PLS regression algorithm [40]. The Bayes theorem was used to create a threshold at the point where the number of false positives and false negatives is minimized [39].

The independent variable X corresponds with the data collected by the sensors, and Y is the dependent categorical variable, a dummy matrix, where the Y value of each class of tuna (unfrozen or frozen-thawed) is expressed in binary code (as 1 s and 0 s, depending on the class of the tuna sample).

The calibration dataset was used to train the models. In order to select the optimal number of latent variables (LV), which should give a low error with the lowest complexity, and to cross-validate the models, a Venetian Blinds cross-validation (CV) with 5 CV groups was performed. Finally, they were validated using the validation dataset.

The performance of the models was evaluated with several metrics: sensitivity, specificity and precision of each class. Sensitivity and specificity are symmetrical parameters in two classes problems. The former describes the model ability to correctly recognize samples belonging to one class while the latter describes the model ability to reject samples of all other classes. Precision measures, in all the samples classified as one class, the fraction that actually belongs to that class. In addition, the error rate (1 minus the arithmetic mean of all class non-error rates) and accuracy (ratio of correct assignments) of each model were computed [39,41].

## 3. Results

### 3.1. Differences between Unfrozen and Frozen-Thawed Samples

The unfrozen samples showed a significantly higher percentage of moisture (68.84 ± 3.32%) than the frozen-thawed samples (63.80 ± 3.72%) (F = 51.28, *p* = 0.00), indicating a water loss phenomenon during the freezing-thawing process. However, despite this water loss, the moisture/protein ratio was statistically different between the control (2.71) and injected samples (2.85) in thawed state (F = 42.32, *p* = 0.00), showing that added water was still present in injected samples after they were frozen and thawed.

### 3.2. Descriptive Statistics of Calibration and Validation Samples

Since the calibration and validation datasets were different for each sensor (the duplex algorithm was applied to the spectral data obtained with each device), the moisture, protein and fat values of each dataset are different.

The mean value between the calibration and validation datasets, expressed in percentages, was very similar for moisture (65.49 ± 4.78 and 65.34 ± 4.93 in BIA; 65.54 ± 4.80 and 65.35 ± 4.69 in TDR; 65.61 ± 4.80 and 64.82 ± 4.64 in NIR, respectively) and protein (23.10 ± 0.97 and 23.05 ± 0.99 in BIA; 23.14 ± 0.99 and 23.22 ± 1.03 in TDR; 23.13 ± 0.99 and 22.94 ± 0.88 in NIR, accordingly). Regarding the fat content, although the differences were bigger (7.94 ± 5.31; 9.67 ± 5.81 in BIA; 8.73 ± 5.82 and 6.89 ± 4.34 in TDR; 8.13 ± 5.27 and 9.30 ± 5.49 in NIR, respectively) the ANOVA analysis shows that they were not statistically significant.

### 3.3. Models Building

The results of the PLS-DA models elaborated for BIA, NIR and TDR are shown in Table 2, Table 3 and Table 4. For each technology, the pre-processing that gave the best performance was chosen.

**Figure 1:**
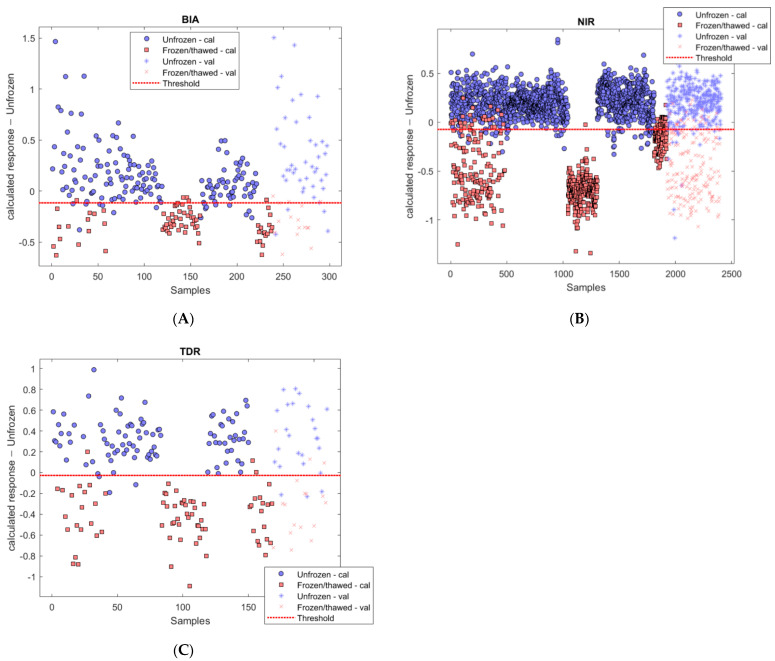
Calculated response for class unfrozen in: BIA (**A**), NIR (**B**) and TDR (**C**). In the calibration set, unfrozen samples are represented with circles and frozen-thawed samples with squares. In the validation set, unfrozen samples are represented with + and frozen-thawed samples with x. “Cal” and “Val” mean samples used for calibration and validation, respectively. BIA: bioelectrical impedance snalysis. NIR: near-infrared spectroscopy. TDR: time domain re-flectometry.

Figure 1 shows the calculated response of the three models for the unfrozen class. As it is a binary classification, samples that do not belong to unfrozen class are classified as the other class (frozen-thawed). The threshold was calculated based on the Bayes theorem, which assumes that the estimated values follow a normal distribution that can be comparable to samples observed in the future [39]. The X-axis shows the number of the sample, and the Y axis represents the probability that each sample has to belong to the unfrozen class.

The PLS-DA classification models showed good performances in the validation data set for the three technologies. In this case, NIR spectroscopy provided the best classification, with an accuracy of 0.91. BIA and TDR outcomes resulted almost equally good, with an accuracy of 0.88 and 0.86, respectively. In general, unfrozen samples had higher values of sensitivity than frozen-thawed samples for BIA, NIR and TDR, respectively (0.91 vs. 0.81; 0.94 vs. 0.86; 0.88 vs. 0.82). The same happens with the precision (0.93 vs. 0.76, 0.92 vs. 0.89 and 0.88 vs. 0.82, accordingly).

**Figure 2:**
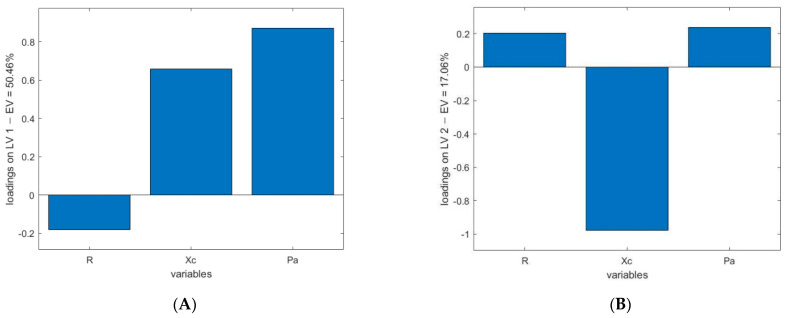
Loadings of LV1 (**A**) and LV2 (**B**) in BIA.

**Figure 3:**
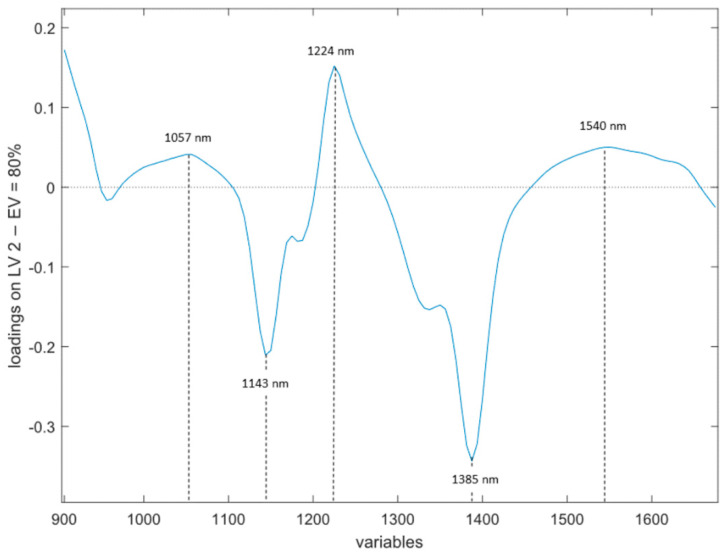
Loading of LV2 in NIR.

**Figure 4:**
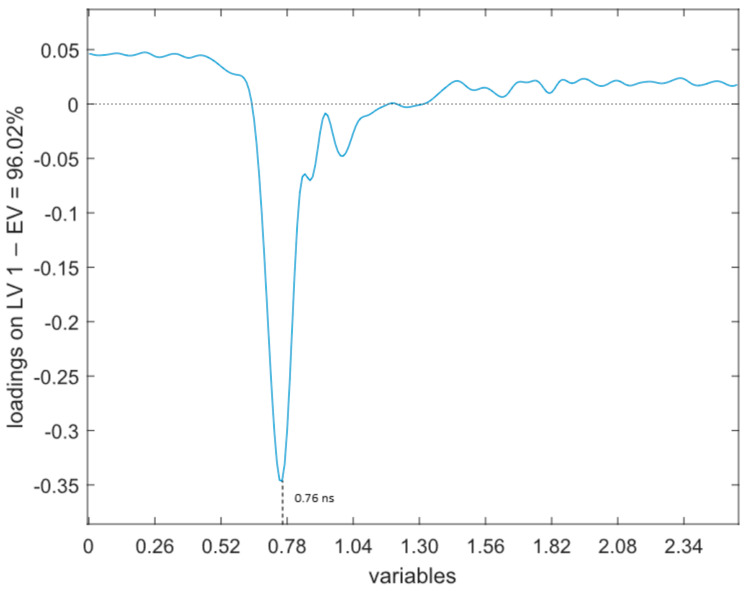
Loading of LV1 in TDR.

The most influential variables for each technology, using the loadings of the models, as represented in Figure 2, Figure 3 and Figure 4, appear to be:

- BIA (Figure 2a,b). Loadings of the first two LVs explain the higher amount of variance (50.46% for latent variable 1 (LV1) and 17.06% for latent variable 2 (LV2)). The loadings of these two LVs reveal that two variables have the higher influence in the model: Pa and Xc.
- NIR (Figure 3). In this case, the LV that retains the higher amount of information is LV2, with an 80.0% explained variance. In this case, an alternating positive and negative pattern is found. Three positive groups of wavelengths are contributing to the model at 980–1100 nm, 1200–1280 nm and 1460–1650 nm, with maximum peaks at 1057 nm, 1224 nm and 1540 nm. The spectral ranges contributing with negative signs are at 1100–1200 nm and 1280–1460 nm, with maximum peaks at 1143 nm and 1388 nm.
- TDR (Figure 4). The loadings of the first LV explain 96.02% of the variance, showing a relevant peak in the region between 0.61 ns and 1.17 ns and a maximum with negative sign at 0.76 ns.

## 4. Discussion

### 4.1. Changes during Frozen-Thawed Process

As it is known, the freezing process applied to fish causes the formation of ice crystals within the muscle tissue. More in detail, when slow freezing rates are applied, large extracellular ice crystals are created, which produces damage in the muscle proteins and cell membranes. On the contrary, during the thawing process, the melting of the ice crystals occurs. This process causes physical and chemical changes in the frozen products, affecting their quality [42]. Generally, the freezing and thawing process contributes to a decrease in the water holding capacity (WHC) of muscles due to the denaturation of the muscle proteins. The empty spaces remaining after the ice crystal melting result in incomplete restoration of the muscle tissues, increasing the drip loss and creating softer textures in muscles, gaps and changes in taste and flavor [43,44].

The destructive analysis (Section 3.1) showed higher moisture in unfrozen than in frozen-thawed samples, as expected due to the decrease in the water holding ability in the frozen-thawed muscle [44]. These analyses also showed differences in the moisture/protein ratio between the control and injected samples, indicating an actual water retention. However, these injections with water and additive solutions did not prevent differentiating unfrozen from thawed samples with both destructive and non-destructive methods.

### 4.2. BIA

The results obtained with BIA are consistent with previous studies related with the use of the technology in the freezing-thawing processes. Vidaček et al. [45] measured sea bass samples subjected to different freezing methods and numbers of freezing cycles using this technology. They found that electrical measurements have the potential to discriminate between unfrozen and frozen-thawed samples. Cox [22] measured albacore tuna with BIA before and after the freezing process, finding changes in the variables Xc and Pa between fresh and thawed fish. However, apart from the previously mentioned works, more focused on the freezing process, this is the first study to use BIA analysis to differentiate unfrozen and frozen-thawed seafood.

BIA measurements (specifically Xc) have also been used to control the integrity of the membranes during the freezing process, showing that the sensor is sensitive to changes in the cell membranes state. Furthermore, some authors have determined that, when freezing at −20 °C, most of the cells would have been destroyed [22,46].

In this study, Figure 2 indicates that the variable Pa has the highest influence in the LV1 and the variable Xc in the LV2. It is in accordance with the changes found by Cox [22] and can be explained by the process of damage on the cell membranes that is described in Section 4.1. The degradation of the membrane during the freezing process affects the integrity of the cells, decreasing the WHC and inducing the drip loss, together with ions that are lost or remain in the extracellular environment. The movement of ions and the change in the extracellular salt concentration make the influence of the variable Xc and, subsequently, the variable Pa higher than the variable R [45].

### 4.3. NIR

Several authors have related the spectral region between 900 and 1400 nm with the vibrational modes of O–H and C–H bonds, which are related to the relaxation of lipid structure and the release of exudates due to the rupture of cell walls and protein denaturation occurring during thawing. This may explain the alternating behavior of the loadings and suggests that the model is using the water/lipid balance between the unfrozen and frozen-thawed samples [47].

On the one hand, the regions contributing to the PLS-DA model with positive values in the loadings seem to be related to the O–H bonds, which might be giving information of water in the fish muscle. The first region (Figure 3) is the one encompassed between 980 and 1100 nm. Sannia et al. [48] related the 960–980 nm region to the moisture content in fish tissues. In our case, the spectral range might have suffered a small shift to the right due to the electrolytes present in the solutions added to some samples, which may have caused changes in the height, width and position of the water absorbance bands [49]. The peak found at 1224 nm has been useful to predict both water and fat content in lamb meat [50]. The last region is found between 1460 and 1650 nm, with a peak at 1540 nm. The wavelength range between 1400 and 1524 nm has been linked to an increase in free water species during thawing [47,48]. However, other authors have related peaks at 1510 nm and 1690 nm with N–H overtones (proteins) in minced beef, though it was difficult to distinguish them due to their closeness to water absorption bands [51]. This overlapping may be explained due to the physical changes that the tissues undergo during the freezing/thawing process. The freezing process alters the capacity to bind water and water distribution [52], causing a denaturation of myofibrillar proteins and decreasing their WHC [28], as it was explained in Section 4.1. When the fish is thawed, the melting of ice crystals of both extracellular water and the water no longer bound to the muscle denatured proteins are converted to free water. On the other hand, peaks in the negative region of the loadings curve were located at 1143 nm and 1388 nm and have been associated with lipids [26,47].

### 4.4. TDR

The results obtained with TDR are in accordance with those of other authors, which showed the potential of this technology to measure seafood quality in relation to freezing processes. Kent et al. [53] investigated the use of time domain reflectometry to differentiate between once and twice frozen-thawed cod (*Gadus morhua*) and inclusively to predict storage time of frozen cod. However, the distinction between fresh and frozen-thawed samples was not addressed. Mendes et al. [54] observed a distinction in the dielectric properties between fresh and frozen-thawed *Octopus vulgaris*, using the same equipment. Nevertheless, the effect of water addition was not investigated simultaneously with the freezing-thawing process.

**Figure 5:**
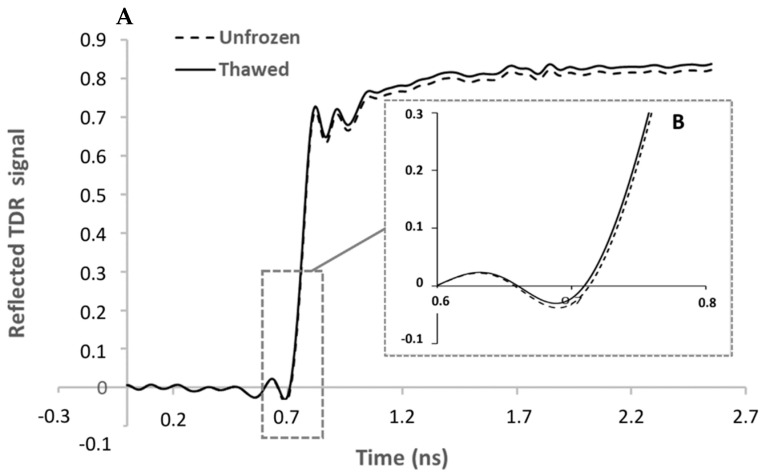
Average value of TDR raw data for unfrozen and thawed samples. (**A**) is the signal of the TDR sensor. (**B**) corresponds with the amplified curve between 0.6 ns and 0.8 ns.

In this study, the LV1 of the model (Figure 4) shows that the region between 0.61 ns and 1.17 ns, with a peak at 0.76 ns, has the greatest contribution for the model, and so to distinguish between unfrozen and thawed tuna samples. According to Fulladosa et al. [55], changes in the signal due to the water content of the sample are mainly expected to be found on the rising edge of the step function, which is located in the region between 0.6 ns and 0.8 ns (Figure 5). This suggests that the sensor is sensitive to the water loss suffered during the freezing-thawing process and that the model is using the water content differences to classify between unfrozen and frozen-thawed samples.

In a previous study with dry-cured hams, the freezing-thawing process (3 days at −18 °C) caused an increase in the normalized reflected TDR signal due to the microstructural damages and the loss of water and ions [56]. This phenomenon also occurs between unfrozen and frozen-thawed raw data in this experiment, although it is more evident above 1.2 ns (Figure 5). As stated by Fulladosa et al. [55], this region is related to changes in the salt content, which would explain why this region, although presenting differences in the raw data, does not have a big contribution in the model to discriminate between fresh and frozen-thawed tuna.

## 5. Conclusions

The findings of this work highlighted the potential of three non-destructive sensors (BIA, NIR and TDR) to discriminate between fresh and frozen-thawed tuna samples which may or may not have added water, a situation that is possible to be found in the fish industry and market. NIR performed the best classification, with an accuracy in the model of 0.91 and an error rate of 0.10 during the validation stage. However, it must be stressed that due to the characteristics of the sensor, more data were acquired and processed. The BIA and TDR sensors also gave good results, giving accuracies of 0.88 and 0.86 and error rates of 0.14 and 0.15, respectively.

This work opens new opportunities to the food industry and/or official control authorities to perform quality control in seafood in order to detect, in a rapid and non-destructive way, whether fish fillets are really unfrozen or have been previously frozen and thawed.

## Figures and Tables

**Table 1:** Number of scans measured with BIA, NIR and RFQ-Scan^®^ equipment.

	Unfrozen		Thawed	
	Non-Injected	Injected	Non-Injected	Injected
BIA	120	90	20	70
NIR	960	720	160	560
TDR	30	90	20	70

BIA: bioelectrical impedance snalysis. NIR: near-infrared spectroscopy. TDR: time domain re-flectometry.

**Table 2:** BIA results.

		Pre-Processing	LV	Error-Rate	Accuracy	Sensitivity	Specificity	Precision
Calibration	Unfrozen	Autoscaling	2	0.08	0.91	0.90	0.93	0.97
	Thawed					0.93	0.90	0.81
CV	Unfrozen			0.10	0.90	0.90	0.90	0.95
	Thawed					0.90	0.90	0.80
Validation	Unfrozen			0.14	0.88	0.91	0.81	0.93
	Thawed					0.81	0.91	0.76

CV: cross-validation. LV: latent variables.

**Table 3:** NIR results.

		Pre-Processing	LV	Error-Rate	Accuracy	Sensitivity	Specificity	Precision
Calibration	Unfrozen	1st derivative (order 2, window 5) + Mean Center	9	0.08	0.94	0.96	0.88	0.95
	Thawed					0.88	0.96	0.91
CV	Unfrozen			0.08	0.94	0.96	0.88	0.95
	Thawed					0.88	0.96	0.90
Validation	Unfrozen			0.10	0.91	0.94	0.86	0.92
	Thawed					0.86	0.94	0.89

**Table 4:** TDR results.

		Pre-Processing	LV	Error-Rate	Accuracy	Sensitivity	Specificity	Precision
Calibration	Unfrozen	SNV + Mean Center	8	0.04	0.96	0.97	0.96	0.97
	Thawed					0.96	0.97	0.96
CV	Unfrozen			0.13	0.87	0.83	0.92	0.93
	Thawed					0.92	0.83	0.81
Validation	Unfrozen			0.15	0.86	0.88	0.82	0.88
	Thawed					0.82	0.88	0.82

## Data Availability

The datasets generated for this study are available on request to the corresponding author.

## References

[1] European Market Observatory for Fisheries and Aquaculture Products (EUMOFA) (2017). EU Consumer Habits Regarding Fishery and Aquaculture Products. https://www.eumofa.eu/documents/20178/84590/EU+consumer+habits_final+report+.pdf/5c61348d-a69c-449e-a606-f5615a3a7e4c.

[2] European Market Observatory for Fisheries and Aquaculture Products (EUMOFA) (2019). The EU Fish Market. https://www.eumofa.eu/documents/20178/314856/EN_The+EU+fish+market_2019.pdf/.

[3] Uddin M., Nollet L.M.L., Toldrá F. (2010). Differentiation of Fresh and Frozen–Thawed fish. Handbook of Seafood and Seafood Product Analysis.

[4] Bozzetta E., Pezzolato M., Cencetti E., Varello K., Abramo F., Mutinelli F., Ingravalle F., Teneggi E. (2012). Histology as a Valid and Reliable Tool to Differentiate Fresh from Frozen-Thawed Fish. J. Food Prot..

[5] Kilinc B., Cakli S., Dincer T., Cadun A. (2009). Effects of phosphates treatment on the quality of frozen-thawed fish species. J. Muscle Foods.

[6] van Ruth S.M., Brouwer E., Koot A., Wijtten M. (2014). Seafood and Water Management. Foods.

[7] (2011). Regulation (EU) No 1169/2011 of the European Parliament and of the Council of 25 October 2011. Off. J. Eur. Union.

[8] FAO (1982). FAO Fisheries Circulars No. 750.

[9] JAS (2000). Notification No. 514 of the Ministry of Agriculture, Forestry and Fisheries.

[10] (2011). Regulation (EU) No 1276/2011 of the European Parliament and of the Council of 8 December 2011. Off. J. Eur. Union.

[11] Reilly A. (2018). Overview of Food Fraud in the Fisheries Sector. FAO Fisheries and Aquaculture Circular. https://www.fao.org/documents/card/en/c/I8791EN/.

[12] Karoui R., Thomas E., Dufour E. (2006). Utilisation of a rapid technique based on front-face fluorescence spectroscopy for differentiating between fresh and frozen–thawed fish fillets. Food Res. Int..

[13] Nott K.P., Evans S.D., Hall L.D. (1999). Quantitative magnetic resonance imaging of fresh and frozen-thawed trout. Magn. Reson. Imaging.

[14] Cheng J.H., Sun D.W., Pu H.B., Chen X., Liu Y., Zhang H., Li J.L. (2015). Integration of classifiers analysis and hyperspectral imaging for rapid discrimination of fresh from cold-stored and frozen-thawed fish fillets. J. Food Eng..

[15] Oehlenschläger J., Luten J.B., Oehlenschläger J., Ólafsdóttir G. (2003). Measurement of freshness quality of fish based on electrical properties. Quality of Fish from Catch to Consumer: Labelling, Monitoring and Traceability.

[16] Kent M., Oehlenschläger J., Rehbein H., Oehlenschläger J. (2009). Measuring Electrical Properties. Fishery Products: Quality, Safety and Authenticity.

[17] Zhao X., Zhuang H., Yoon S.C., Dong Y., Wang W., Zhao W. (2017). Electrical Impedance Spectroscopy for Quality Assessment of Meat and Fish: A Review on Basic Principles, Measurement Methods, and Recent Advances. J. Food Qual..

[18] Karoui R., Lefur B., Grondin C., Thomas E., Demeulemester C., Baerdemaeker J.D., Guillard A.S. (2007). Mid-infrared spectroscopy as a new tool for the evaluation of fish freshness. Int. J. Food Sci. Technol..

[19] Sivertsen A.H., Kimiya T., Heia K. (2011). Automatic freshness assessment of cod (*Gadus morhua*) fillets by Vis/Nir spectroscopy. J. Food Eng..

[20] Velioğlu H.M., Temiz H.T., Boyaci I.H. (2015). Differentiation of fresh and frozen-thawed fish samples using Raman spectroscopy coupled with chemometric analysis. Food Chem..

[21] Karoui R., Blecker C. (2011). Fluorescence Spectroscopy Measurement for Quality Assessment of Food Systems—A Review. Food Bioprocess Technol..

[22] Cox M.K. (2015). Bioelectrical Impedance Analysis Measures of Body Composition and Condition, and Its Sensitivity to the Freezing Process. J. Aquat. Food Prod. Technol..

[23] Zhou J.J., Wu X.Y., Chen Z., You J., Xiong S.B. (2019). Evaluation of freshness in freshwater fish based on near infrared reflectance spectroscopy and chemometrics. LWT Food Sci. Technol..

[24] Huang H., Yu H., Xu H., Ying Y. (2008). Near infrared spectroscopy for on/in-line monitoring of quality in foods and beverages: A review. J. Food Eng..

[25] Nicolaï B.M., Beullens K., Bobelyn E., Peirs A., Saeys W., Theron K.I., Lammertyn J. (2007). Nondestructive measurement of fruit and vegetable quality by means of NIR spectroscopy: A review. Postharvest Biol. Technol..

[26] Reis M.M., Martínez E., Saitua E., Rodríguez R., Pérez I., Olabarrieta I. (2017). Non-invasive differentiation between fresh and frozen/thawed tuna fillets using near infrared spectroscopy (Vis-NIRS). LWT Food Sci. Technol..

[27] Uddin M., Okazaki E. (2004). Classification of Fresh and Frozen-thawed Fish by Near-infrared Spectroscopy. J. Food Sci..

[28] Kent M., Daschner F., Rehbein H., Oehlenschläger J. (2009). Time domain spectroscopy. Fishery Products: Quality, Safety and Authenticity.

[29] Jha S.N., Narsaiah K., Basediya A.L., Sharma R., Jaiswal P., Kumar R., Bhardwaj R. (2011). Measurement techniques and application of electrical properties for nondestructive quality evaluation of foods—A review. J. Food Sci. Technol..

[30] Büning-Pfaue H. (2003). Analysis of water in food by near infrared spectroscopy. Food Chem..

[31] Kyle U.G., Bosaeus I., De Lorenzo A.D., Deurenberg P., Elia M., Gómez J.M., Heitmann B.L., Kent-Smith L., Melchior J.C., Pirlich M. (2004). Bioelectrical impedance analysis—Part I: Review of principles and methods. Clin. Nutr..

[32] Gudjónsdóttir M., Karlsdóttir M.G., Arason S., Rustad T. (2013). Injection of fish protein solutions of fresh saithe (*Pollachius virens*) fillets studied by low field Nuclear Magnetic Resonance and physicochemical measurements. J. Food Sci. Technol..

[33] Saint-Denis T., Goupy J. (2004). Optimization of a nitrogen analyser based on the Dumas method. Anal. Chim. Acta.

[34] AOAC (2005). Official Methods of Analysis of the Association of Official Analytical Chemists International.

[35] Bligh E.G., Dyer W.J. (1959). A rapid method of total lipid extraction and purification. Can. J. Biochem. Physiol..

[36] Schimmer O., Knöchel R., Thakur K. (2003). A hand-held TDR-system with a fast system-rise time and a high resolution bandwidth for moisture measurements in the microwave frequency range. Proceedings of the 5th International Conference on Electromagnetic Wave Interaction with Water and Moist Substances.

[37] Rinnan Å., Van Den Berg F., Engelsen S.B. (2009). Review of the most common pre-processing techniques for near-infrared spectra. TrAC Trends Anal. Chem..

[38] Snee R.D. (1977). Validation of Regression Models: Methods and Examples. Technometrics.

[39] Ballabio D., Consonni V. (2013). Classification tools in chemistry. Part 1: Linear models. PLS-DA. Anal. Methods.

[40] Wold S., Sjöström M., Eriksson L. (2001). PLS-regression: A basic tool of chemometrics. Chemom. Intell. Lab. Syst..

[41] Davis J., Goadrich M., Cohen W., Moore A. (2006). The relationship between Precision-Recall and ROC curves. Proceedings of the 23rd International Conference on Machine Learning.

[42] Hassoun A., Shumilina E., Di Donato F., Foschi M., Simal-Gandara J., Biancolillo A. (2020). Emerging Techniques for Differentiation of Fresh and Frozen–Thawed Seafoods: Highlighting the Potential of Spectroscopic Techniques. Molecules.

[43] Leygonie C., Britz T.J., Hoffman L.C. (2012). Impact of freezing and thawing on the quality of meat: Review. Meat Sci..

[44] Nakazawa N., Okazaki E. (2020). Recent research on factors influencing the quality of frozen seafood. Fish. Sci..

[45] Vidaček S., Medić H., Botka-Petrak K., Nežak J., Petrak T. (2008). Bioelectrical impedance analysis of frozen sea bass (*Dicentrarchus labrax*). J. Food Eng..

[46] Davalos R., Rubinsky B. (2004). Electrical Impedance Tomography of Cell Viability in Tissue with Application to Cryosurgery. J. Biomech. Eng..

[47] Pennisi F., Giraudo A., Cavallini N., Esposito G., Merlo G., Geobaldo F., Acutis P.L., Pezzolato M., Savorani F., Bozzetta E. (2021). Differentiation between Fresh and Thawed Cephalopods Using NIR Spectroscopy and Multivariate Data Analysis. Foods.

[48] Sannia M., Serva L., Balzan S., Segato S., Novelli E., Fasolato L. (2019). Application of near-infrared spectroscopy for frozen-thawed characterization of cuttlefish (*Sepia officinalis*). J. Food Sci. Technol..

[49] Laub-Ekgreen M.H., Martinez-Lopez B., Jessen F., Skov T. (2018). Non-destructive measurement of salt using NIR spectroscopy in the herring marinating process. LWT Food Sci. Technol..

[50] Kamruzzaman M., ElMasry G., Sun D.W., Allen P. (2012). Non-destructive prediction and visualization of chemical composition in lamb meat using NIR hyperspectral imaging and multivariate regression. Innov. Food Sci. Emerg. Technol..

[51] Morsy N., Sun D.W. (2013). Robust linear and non-linear models of NIR spectroscopy for detection and quantification of adulterants in fresh and frozen-thawed minced beef. Meat Sci..

[52] Ballin N.Z. (2010). Authentication of meat and meat products. Meat Sci..

[53] Kent M., Knöchel R., Daschner F., Schimmer O., Oehlenschläger J., Mierke-Klemeyer S., Barr U.K., Floberg P., Tejada M., Huidobro A. (2004). Time domain reflectometry as a tool for the estimation of quality in foods. Int. Agrophys..

[54] Mendes R., Schimmer O., Vieira H., Pereira J., Teixeira B. (2018). Control of abusive water addition to *Octopus vulgaris* with non-destructive methods. J. Sci. Food Agric..

[55] Fulladosa E., Duran-Montgé P., Serra X., Picouet P., Schimmer O., Gou P. (2013). Estimation of dry-cured ham composition using dielectric time domain reflectometry. Meat Sci..

[56] Rubio-Celorio M., Garcia-Gil N., Gou P., Arnau J., Fulladosa E. (2015). Effect of temperature, high pressure and freezing/thawing of dry-cured ham slices on dielectric time domain reflectometry response. Meat Sci..

